# Examining retail purchases of cigarettes and nicotine replacement therapy in Finland

**DOI:** 10.18332/tid/108537

**Published:** 2019-05-03

**Authors:** David S. Timberlake, Johanna Joensuu, Terhi Kurko, Arja H. Rimpelä, Jaakko Nevalainen

**Affiliations:** 1 Faculty of Social Sciences, Tampere University, Tampere, Finland; 2 Program in Public Health, University of California, Irvine, United States; 3 The Social Insurance Institute of Finland, Helsinki, Finland; 4 PERLA - Tampere Centre for Childhood, Youth and Family Research, Tampere University, Tampere, Finland; 5 Department of Adolescent Psychiatry, Tampere University Hospital, Tampere, Finland

**Keywords:** nicotine replacement therapy, cigarettes, tobacco endgame, time-series data, retail purchases

## Abstract

**INTRODUCTION:**

Finland’s success in achieving the goal of its tobacco endgame largely depends on rectifying deficiencies in the delivery of smoking cessation services. One such weakness, which has not been documented with empirical data, is misuse of nicotine replacement therapy (NRT). This study’s objective was to examine purchase patterns of NRT for estimating improper use of the medication. The study was based on the assumption that duration of a purchase episode is indicative of either proper use or misuse of NRT.

**METHODS:**

The participants (n=728), who purchased at least one NRT product in 2016 (mostly gum/lozenge), were selected through enrollment in a large customer loyalty program in Finland (LoCard). Participants were categorized into one of five groups according to their longest purchase episode of NRT, defined by purchases made in consecutive, 4-week intervals.

**RESULTS:**

Most participants, who did not adhere to NRT guidelines, either purchased the medication for too short (≤4 weeks, 63.5%) or too long (>24 weeks, 13.2%) of a purchase episode. Median purchases of NRT in the first month of use were one and four in the former and latter, respectively. In contrast to other groups, persistent users (>24 weeks) did not curtail purchases of NRT across several 4-week intervals, suggesting potential for dependence on NRT.

**CONCLUSIONS:**

The observation that most purchase episodes were terminated prematurely is consistent with surveys reporting widespread NRT misuse. Given uncertainty of greater regulation of NRT sales through legislation, it would be prudent for Finnish retailers to promote proper use of the therapy.

## INTRODUCTION

Providing nicotine replacement therapy (NRT) to smokers without a doctor’s prescription, via an over-the-counter (OTC) purchase, was a significant initiative to increase accessibility and use of the proven therapy^1^. Critics argue that easier access to the medication reduces the interaction between patient and provider, which is critical for a successful quit attempt. Others contend that even after prescribing medication, physicians seldom provide proper cessation counseling^2,3^. In countries such as Finland, pharmacists provided counseling because OTC sales of NRT were initially restricted to pharmacies. But, the Finnish Parliament approved a law that deregulated the NRT market in 2006, allowing retail sales in grocery stores, kiosks and gasoline stations^4^. Prices of NRT products, which declined by 15% after deregulation, were the least expensive in hypermarkets, followed by supermarkets and pharmacies^5^. Consequently, sales of NRT increased dramatically in Finland^6^, but not in pharmacies^7^ where consultation occurs on the proper use of the medication.

The initial concern about liberalizing the NRT market focused on smokers’ potential for prolonging use of the medication beyond the 3-month period set by the US Food and Drug Administration (FDA). This concern was largely dispelled by studies reporting that only 6% of participants purchased and used NRT for an extended period of at least six months^8,9^. It is believed that most smokers prolong their use of NRT in order to quit or reduce smoking rather than satiate an addiction. Consequently, researchers advocated that guidelines for duration of NRT use be extended beyond the 3-month maximum^10,11^. A more pressing issue relates to the majority of smokers who prematurely terminate the use of NRT^12^. Using a longitudinal design, Zhang et al.^12^ reported that smokers who used NRT for less than four weeks had a decreased likelihood of achieving abstinence relative to the non-NRT users (OR=0.51, 95% CI: 0.38–0.67). The ineffectiveness of using NRT for such a brief period is concerning because studies, such as the ITC Four-Country Survey^13^, indicate that OTC NRT is prematurely terminated among the majority of users. Balmford et al.^13^ reported that 62.9% and 76.3% of users of OTC NRT had only used the medication for ≤4 weeks and ≤8 weeks, respectively. Such studies, however, are limited by the use of self-reported data that cannot be verified. The alternative of analyzing time-series purchases is one means of obviating the inaccuracies that occur with survey data. The current study used retail data for estimating the percentage of NRT users who purchased the medication for too short or too long of a purchase episode.

An examination of retail purchases is particularly needed in light of Finland’s goal to end the use of tobacco by 2030 (i.e. <5% prevalence). The Roadmap to a Tobacco–Free Finland specifies several steps in treating tobacco dependence^14^ as part of the larger goal of rectifying deficiencies in smoking cessation services. As evidence of one such deficiency, Finland received the low score of 5/10 points for tobacco treatment on the 2016 Tobacco Control Scale^15^. One notable recommendation in the Roadmap is to reimburse smokers for medications and remove Section 54a from the Medicines Act, the latter permitting sales of OTC NRT in retail outlets (e.g. grocery stores). The absence of purchase data, which predates the deregulation of Finland’s NRT market in 2006, precludes assessment of whether purchases from retail outlets contribute to greater misuse of NRT relative to purchases from pharmacies. The current study is neither designed to test effectiveness of regulated versus deregulated NRT markets, nor is it designed to examine patterns of use and motives for purchasing NRT. Instead, this study aims to examine the duration of NRT purchase episodes for estimating the duration of episodes of NRT use. The goal is to verify the extent of NRT misuse^13^ through estimation of purchase episodes that are terminated prematurely. The findings could serve as baseline estimates in the case of future policy change, and provide retailers information that could facilitate efforts to increase customers’ adherence to NRT guidelines.

## METHODS

### Sample selection

Participants of this study were selected through their enrollment in a customer loyalty program headed by the S Group, a commercial enterprise possessing more than 45% of the market share in Finland^16^. The S Group’s large market share minimizes but does not negate the possibility that study participants could have purchased cigarettes and NRT from other sources. Using a database containing customers’ email addresses, the S Group contacted members of the customer loyalty program in the HOK–Elanto retail co-operative in southern Finland. The email inquiry sought members’ consent to release basic demographic information (age, gender, postal code) and purchases made in the year 2016. Release of the data, which excluded personal identifiers, was intended for research purposes and approved by the ethics committee of the University of Helsinki Review Board in the humanities and social and behavioral sciences. Approximately 5% of members of the loyalty program consented to the data release, yielding a sample of 13274 customers. Compared to the general population in southern Finland, consenting members of the loyalty program were predominantly female (about 68%), middle-aged (about 46 years old), and more educated, which can be attributed to higher participation rates^16^. This is evidenced by the statistic indicating that 56% versus 33% of consenting loyalty members and the general population, respectively, had a university degree^17^. Our final analytic sample, which excluded S Group personnel, consisted of customers who purchased at least one NRT product in the year 2016 (n=728).

### Measures

The measures were the time, date, and expenditures of cigarettes and NRT in the year 2016. Categories of NRT consumers were based on the purchase of one or more products in consecutive 4-week intervals. The categories, which were based on clinical guidelines and empirical findings^12^, correspond to: the early terminators (≤4 weeks of use); those who used NRT for the minimum period (5–8 weeks); those who used NRT for the standard recommended period (9–12 weeks); those who extended their use of NRT according to revised recommendations (13–24 weeks)^10^; and those who exceeded the maximum period of NRT use (>24 weeks). The study participants were categorized into one of the five mutually exclusive groups according to their longest purchase episode, absent a purchase lapse exceeding 28 days. For example, if a participant had purchased NRT on 5 February, 21 February and 16 March, and made a subsequent purchase on 9 June, then the participant was classified as having purchased NRT for 5–8 weeks of use (range: 29–56 days). For those participants who had multiple but equal purchase episodes, the longest episode in days was selected. An interruption in the purchase of NRT exceeding one month was used previously in signifying a new episode of use^9^. Our classification scheme did not account for those who made bulk purchases of NRT for use over a lengthy time period. Furthermore, the classification scheme was not based on incident purchases, and, thus, does not necessarily represent individuals who used NRT for the first time. For example, an early terminator who made a purchase in January 2016 may have purchased NRT throughout the latter part of 2015.

Based on sales data from the S Group, nicotine gum and lozenge constituted 96.9% of the total sales volume of all nicotine replacement products. Sales of the nicotine patch, which were low and unchanging from 2006 (deregulation) through 2013^6^, were slightly higher in pharmacies compared to other outlets. Given its low overall volume, it is unlikely that the nicotine patch was supplemented with copious purchases of nicotine gum and lozenge. Data released by the S Group included neither the product type (e.g. gum, lozenge, patch) nor product characteristics such as brand, package size, or dosage in milligrams. Expenditures of NRT were available for analysis, but, overlapped too much to differentiate product types. Thus, we opted not to extrapolate package size from expenditure for estimating the supply of NRT for use over a designated period. Instead of extrapolating package size, we used consecutive purchases over 4-week intervals as a measure of duration of NRT use, which does not imply that the purchase(s) provided a sufficient supply of NRT for any given month. Although expenditures of NRT were not used in extrapolating package sizes, they were summed over intervals as a descriptive way of illustrating purchase differences among the five groups of NRT consumers.

### Data analysis

The data analyses entailed the use of paired and non–paired statistical tests. Non-parametric methods were employed due to the skewed distributions of purchases and expenditures of NRT and cigarettes. The Kruskal–Wallis test on ranks, analogous to a one-way ANOVA, was used for comparing purchases and expenditures across the five groups of NRT consumers. One notable comparison across groups was the number of NRT purchases made in the first four weeks of a purchase period. The Wilcoxon signed rank test was used for testing differences within pairs across time periods, by NRT consumer group. Purchases in the first four weeks served as the baseline for comparisons to purchases made in subsequent 4-week intervals. The within-pair tests facilitated assessment of whether NRT purchases stayed the same or diminished following the first four weeks of a purchase episode.

## RESULTS

### Characteristics of NRT consumers

The 728 participants made a total of 9300 NRT and 21601 cigarette purchases in 2016. Most purchases of NRT and cigarettes occurred on Friday, making up 16.2% and 17.4% of total sales, respectively. A higher per cent of females versus males (66.9% vs 57.0%) and young versus the old (72.9% vs 61.6%) prematurely terminated use of NRT. Tests of independence indicated a statistically significant association between NRT consumer group and sex (χ^2^_(4)_=10.8; p=0.03), but not age group (χ^2^_(8)_=10.5; p=0.3) (Table 1).

**Table 1:** Characterizing consumers of nicotine replacement therapy (NRT) by their demographics and purchases of NRT and cigarettes in 2016 (n=728 )

	*All groups*	*Categories of NRT Consumers*					*Test statistic*
		*Early termination*	*Minimum*	*Recommended*	*Extended*	*Exceeded*	
Sample size	728	462	66	43	61	96	
Duration of NRT use defining group^a^		≤4 weeks	5–8 weeks	9–12 weeks	13–24 weeks	25–52 weeks	
**Demographics**							
**Sex** (%)							χ^2^_(4)_=10.8*
Female	64.8	68.4	51.5	58.1	55.7	65.6	
Male	35.2	31.6	48.5	41.9	44.3	34.4	
**Age group** (%)							χ^2^_(8)_=10.5
<30^b^	9.6	11.0	10.6	4.6	9.8	4.2	
≥50 years	31.9	31.0	33.3	23.3	41.0	33.3	
**NRT Purchases**							
Median purchases/ year	3	1	10	12	23	49	χ^2^_(4)_ ^f^=530***
Median purchases/ period^c^	2	1	4	6	13	43.5	χ^2^_(4)_ ^f^=578***
Purchase gap in days^c,d^	9	12	12.7	12	8	6	χ^2^_(4)_ ^f^=82.5***
**NRT Expenditures** (€)							
Median/year	32.3	13.6	90.1	155.7	233.3	633.0	χ^2^_(4)_ ^f^=466***
Median/period^c^	21.6	9.1	40.6	69.4	145.7	585.1	χ^2^_(4)_ ^f^=480***
**Cigarette Purchases & Expenditure**							
% Purchased in 2016	67.6	71.6	65.1	67.4	67.2	50.0	χ^2^_(4)_=17.2**
Median purchases/ year^e^	24	27	34	18	17	11	χ^2^_(4)_ ^f^=4.6
Median expenditures (€) /year^e^	180.4	211.9	224.0	105.5	105.2	91.4	χ^2^_(4)_ ^f^=7.2

a Categories are defined according to purchases made in consecutive 4-week intervals (see Methods)

b Includes small number of adolescents (n=18)

c Corresponds to the period defining each group

d Median was calculated from the distribution of medians of individual participants, excluding those who purchased NRT only one time

e Limited to those who made a cigarette purchase in 2016 (n=492)

f Kruskal-Wallis test for non-parametric data (one-way ANOVA on ranks).

* p<0.05

** p<0.01

*** p<0.0001.

The early terminators, who did not make consecutive purchases across 4-week intervals, composed almost two-thirds of the entire sample. Of the NRT products, 15.2% and 11.6% were purchased by the early terminators during January 2016 and the other eleven months of the year (χ^2^_(1)_=7.8; p=0.005), respectively, suggesting that several early terminators may have purchased the medication as part of a New Year’s resolution. Among the early terminators (n=462), 237 had purchased NRT only one time with a median expenditure of €6.6 (range: €2 – €30.1). These onetime purchasers of NRT frequently purchased cigarettes as evidenced by a high median number of cigarette purchases (31) and cigarette expenditures (€251.8).

The participants who exceeded NRT recommendations (>24 weeks, n=96), referred to as persistent users, had the fewest cigarette purchases due in part to the large per cent (50%) of users who did not make a single cigarette purchase in 2016. The persistent users had a median purchase gap of NRT that was half the value observed in those who bought NRT over the recommended period (6 vs 12; for all groups, χ^2^_(4)_=82.5; p<0.0001). The purchase gaps were assessed within periods of consecutive NRT purchases (gaps ≤28 days), thus negating the possibility that large gaps could be attributed to a later purchase of NRT. Almost 5% of the entire sample (n=35) had purchased NRT for 13 consecutive 4-week intervals in 2016.

### Purchases of NRT over time

For the first month of use, purchases of NRT varied significantly across the five groups of consumers (χ^2^_(4)_=416.9; p<0.0001), with the median purchase ranging from one to four in the early terminators and persistent users, respectively (Table 2). Similar purchase patterns were observed across the NRT consumer groups for the subsequent 4-week intervals. In examining within-pair differences, we observed statistically significant declines in NRT purchases from baseline (i.e. first four weeks of a purchase episode) to the subsequent 4-week intervals in the groups using NRT over the minimum, recommended, and extended time periods. For example, those who followed minimum recommendations had a median of three purchases in the first month of use, but only 1.5 purchases in weeks five through eight (S=624.5; p<0.001). Declines following the first four weeks of purchasing NRT were evident for every group with exception those who exceeded the guidelines. The latter had a median of four purchases in weeks 1–4, weeks 5–8, weeks 9–12, and weeks 13–16. By weeks 25–28, a significant decline in NRT purchases from baseline had been observed (S=760.5; p<0.001). Yet, the persistent users’ purchases and expenditures were rather stable over the whole period.

**Table 2:** A comparison of the quartiles of NRT expenditures and quartiles of number of NRT purchases (in parenthesis) across groups of consumers by four-week periods of NRT use

*4-Week Period of NRT Use*	*Percentile*	*Categories of NRT Consumers*				
		*Early termination ≤4 weeks (n=462 )*	*Minimum 5–8 weeks (n=66 )*	*Recommended 9–12 weeks (n=43 )*	*Extended 13–24 weeks (n=61 )*	*Exceeded 25–52 weeks (n=96 )*
Weeks 1–4***	25%	5.2€ (1)^a^	15.2€ (2)	17.7€ (2)	21.7€ (2)	29.8€ (3)
	50%	9.1€ (1)	23.3€ (3)	35.4€ (3)	39.2€ (3)	56.0€ (4)
	75%	20.1€ (2)	44.7€ (4)	52.0€ (4)	55.3€ (5)	89.7€ (6)
Weeks 5–8***	25%	NA^b^	6.6€ (1)	10.0€ (1)	16.8€ (2)	32.6€ (3)
	50%	NA	15.8€ (1.5)	19.9€ (2)	30.2€ (3)	55.5€ (4)
	75%	NA	34.0€ (2)	43.1€ (3)	43.7€ (4)	83.8€ (6.5)
	Signed rank test		S=624.5**	S=206.5**	S=157*	**S=77.5**
Weeks 9–12***	25%	NA	NA	10.0€ (1)	15.0€ (2)	30.0€ (3)
	50%	NA	NA	18.5€ (1)	29.4€ (2)	50.4€ (4)
	75%	NA	NA	30.0€ (2)	43.2€ (4)	85.8€ (6)
	Signed rank test			S=267**	S=349**	**S=321.5**
Weeks^c^ 13–16***	25%	NA	NA	NA	14.9€ (2)	26.0€ (3)
	50%	NA	NA	NA	29.9€ (2)	53.7€ (4)
	75%	NA	NA	NA	38.6€ (3)	88.5€ (6)
	Signed rank test				S=316.5**	**S=175**
Weeks^c^ 25–28	25%	NA	NA	NA	NA	20.8€ (2)
	50%	NA	NA	NA	NA	47.4€ (3)
	75%	NA	NA	NA	NA	72.7€ (5)
	Signed rank test					S=760.5**

a Each cell represents the quartile 1st (25%), 2nd (50%), or 3rd (75%) of NRT expenditures in euros and quartile of number of NRT purchases (in parenthesis)

b Not applicable

c The 4-week period was selected because everyone within the respective NRT group had purchased NRT during the specified period.

*** p<0.0001 corresponds to a Kruskal–Wallis test on ranks of NRT purchases across groups of consumers;

** p<0.001 and

* p<0.05 correspond to the Wilcoxon signed rank test of NRT purchases across 4-week periods, using the first four weeks of a purchase episode as the baseline comparison.

### Concurrent purchases of cigarettes and NRT

**Figure 1:**
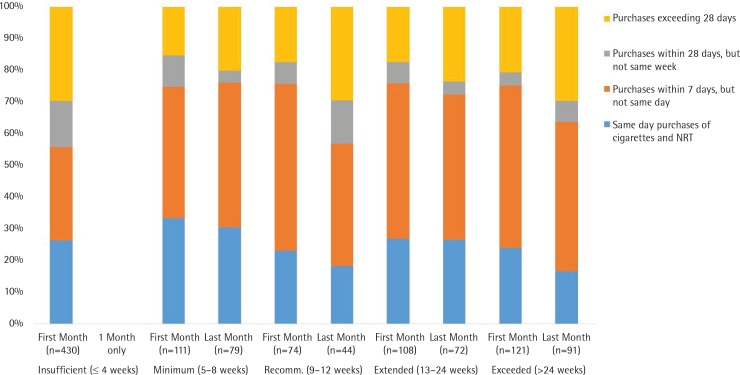
Concurrent purchases of cigarettes and NRT in the first month (n=844 purchases) and last month (n=286 purchases) of a purchase episode

The percentage of concurrent purchases of cigarettes and NRT in the first and last month of a purchase episode, shown in Figure 1, excludes the participants who never purchased cigarettes in 2016. Concurrent purchases were coded in terms of pairs of consecutive purchases of the two products. For example, if a customer purchased a cigarette pack on day 5, another cigarette pack on day 17, and NRT on day 20, then a single concurrent purchase would be coded as having occurred within the same week. In this way, the coding scheme selected the shortest period between a cigarette and NRT purchase.

In their single month of NRT use, the early terminators had a lower percentage of concurrent purchases relative to other groups, which occurred despite frequent purchases of cigarettes throughout the year. Concurrent purchases on the same day or same week, which occurred frequently for the other groups, changed slightly among those who purchased NRT over minimum and extended periods. In contrast, the decline in the percentage of concurrent purchases on the same day/week was more evident for those who followed recommendations (75.7% to 56.8%) and those who exceeded recommendations (75.2% to 63.7%).

## DISCUSSION

The study’s findings suggest that most consumers in Finland are not purchasing NRT over a sufficient period of time. Our estimate of those who purchased NRT for four weeks or less (63.5%) is remarkably similar to use of the medication over the same period reported from the ITC Four–Country Survey (62.9%)^13^. The current study also revealed that the amount of NRT purchased in the first four weeks of a purchase episode was very small in those who terminated early relative to those who proceeded to purchase NRT over lengthier periods. The correlation between amount and duration of NRT purchases could be indicative of success or failure of a cessation attempt^18^.

As reported in other studies^13,19^, the two most commonly cited explanations for prematurely terminating NRT use, relapse to smoking and medication side effects, are legitimate reasons according to manufacturers’ guidelines. A third explanation may be traced to smokers’ concerns about becoming dependent on NRT. Some smokers from Finland, who participated in an online forum (i.e. STUMPPI quitline)^20^, advocated the strict and limited use of NRT in an effort to avoid nicotine dependence. Yet, the same smokers acknowledged that such practice could lead to an unsuccessful quit attempt. In our study, it is likely that many early terminators failed to quit smoking as a function of their frequent cigarette purchases and dependence on nicotine. A fourth explanation for early termination could be traced to improper NRT use arising from lack of instruction and guidance. One study reported that among US smokers who used nicotine gum, only 58% read any of the product inserts^21^.

Our estimate of the NRT purchases exceeding 24 weeks (13.2%) was higher than expected. The most likely explanation is that the US study, which reported 6% of participants who purchased NRT for a minimum of six months^9^, estimated incidence of persistent use, whereas, our study estimated prevalence of persistent use. Some persistent users identified in our study in 2016 probably used NRT throughout 2015, leading to a higher estimate than that reported by Shiffman et al.^9^. Another explanation for our higher estimate is the European guideline recommending the extended use of NRT^22^. In contrast, the study conducted by Shiffman et al.^9^ was conducted prior to the US FDA’s recommendation to extend duration of NRT use^10^. It is notable that 71.6% versus 50% of the early terminators and persistent users, respectively, had purchased cigarettes in 2016. While we do not know the smoking status of those who did not purchase cigarettes, it is likely that most were former smokers who replaced their habit with pharmaceutical nicotine. Current knowledge suggests that persistent use of NRT is less harmful than smoking cigarettes. Unlike other groups in our study, the persistent users did not curtail their use of NRT for several weeks following initiation of a purchase episode. While this is a concern, dependence on oral NRT is an uncommon occurrence^23^.

In lieu of an amendment to the Medicines Act, retailers could take a number of actions to increase customers’ adherence to NRT guidelines. They could, for example, increase customers’ exposure to the guidelines through the dissemination of more instructional brochures. Another option, which is currently being discussed by the S Group, is an online database of purchases of customers enrolled in the loyalty program. At the moment, consideration is being given to tracking grocery purchases for improving the health and nutrition of customers. If such a database were to be developed, then NRT purchases could be readily added along with recommendations for increasing customers’ proper use of the medication. An online feature would be particularly useful because of the high per cent of young customers (<30 years old) who use the Internet but do not properly use NRT. While any one of these retailer initiatives could modify consumer behavior, they are not proven as effective interventions.

### Limitations

The time-series analysis of retail purchases of cigarettes and NRT minimized the measurement error that frequently occurs in survey data. Yet, there are limitations that need to be acknowledged with regard to using purchase data. First, the cigarette and NRT purchases could have been used by someone other than the purchaser, such as a family member. Data indicating more than one smoker per smoking household^9^ suggest the possibility of multiple quit attempts within the same household. Consequently, single purchasers of NRT within such households could have inflated our estimate of NRT purchases per customer. The second limitation is that a loyalty member may have purchased NRT in an S Group market, but purchased cigarettes elsewhere (or vice versa); consequently, neither smoking status nor prevalence of NRT use could be determined. Third, our categorization of NRT consumer groups was based on purchase episodes in 2016, not purchase incidents. This limitation precluded us from estimating an individual’s risk of becoming a persistent user of NRT. We could not determine if the longest purchase episode of NRT occurred in 2016, or some period prior to 2016. Further, we could not determine if an individual who purchased NRT in the latter part of 2016 continued to purchase NRT throughout 2017.

An additional limitation was the inability to assess whether the study’s findings can be generalized from the sample of loyalty members who purchased NRT to the population of NRT consumers in southern Finland. One surrogate measure for purchasing NRT is smoking status. Yet, the absence of data on smoking status in the sample and population precluded us from weighting our estimates according to population proportions of smokers and non-smokers. Since the highly educated were oversampled in our study, it is likely that any such weighting scheme would have yielded a proportion of early terminators of NRT that exceeded the proportion reported in the current study. Yet, in the absence of a weighting scheme, the estimate for early termination of therapy was already inflated by the higher percentage of female customers (who were oversampled) versus male customers who terminated purchases of NRT prematurely. In contrast, Balmford et al.^13^ reported that female smokers had greater odds, though nonsignificant, than male smokers of completing a course of treatment (OR=1.24, 95% CI: 0.95–1.61).

The absence of product type (e.g. gum, lozenge) and product characteristics (e.g. package size) precluded an accurate assessment of whether a consumer purchased a sufficient amount of NRT to cover a 4-week period. The alternative of using more detailed data from Nielsen Company’s Homescan Consumer Panel was not an option because of the insufficient number of participating households in Finland (n=5000; *pers. comm.* Ethan Markovitz, Manager, Client Solutions, Nielsen). Even if more detailed data were available, such information would not have accounted for the variability in daily use of oral NRT^9^.

## CONCLUSIONS

This study supports findings from survey data indicating that most users of NRT terminate the therapy prematurely. In the latest proposal to end tobacco use in Finland by the year 2030, a working group commissioned by the Ministry of Social Affairs and Health recommended amending the Medicines Act for greater monitoring of the distribution channels of OTC NRT^24^. Yet, given the uncertainty of any amendment to the Act, it would be prudent for retailers to take steps to increase customers’ knowledge of proper NRT use and awareness of NRT purchases.
